# Breastfeeding in women with systemic lupus erythematosus: results from a Norwegian quality register

**DOI:** 10.1186/s13006-023-00576-y

**Published:** 2023-08-01

**Authors:** Maylinn Bjerkaas Hanssen, Agnete Malm Gulati, Hege Koksvik, Marianne Wallenius

**Affiliations:** 1 Department of Neuromedicine and Movement Science, Norwegian University of Science and Technology, Trondheim, Norway; 2 Department of Rheumatology, St. Olavs University Hospital, Trondheim, Norway; 3 Office of Medical Education, Norwegian University of Science and Technology, Trondheim, Norway; 4 Department of Rheumatology, The Norwegian National Advisory Unit On Pregnancy and Rheumatic Diseases (NKSR), St. Olavs University Hospital, Trondheim, Norway

## Abstract

**Background:**

Knowledge on breastfeeding among women with systemic lupus erythematosus (SLE) is sparse. We wanted to identify the frequency of breastfeeding in SLE, and to compare breastfeeding women with SLE to non-breastfeeding women to examine possible differences in disease characteristics and self-reported health data between the groups.

**Methods:**

Prospective data on women with SLE from RevNatus, a consent-based Norwegian nationwide quality register was used for this study. Data were collected during January 2016 to September 2021. We used data registered at inclusion when planning pregnancy or in 1^st^ trimester, and 6 weeks, 6 and 12 months after delivery. Breastfeeding and non-breastfeeding patients were compared according to demographic, serological and obstetric data as well as disease activity, medication, self-reported pain, and fatigue.

**Results:**

A total of 114 pregnancies in 101 SLE women were included in the analysis. A majority of the women (78%) breastfed six weeks postpartum. Six and 12 months after delivery, breastfeeding rates were 54% and 30% respectively. Six weeks postpartum, non-breastfeeding women showed higher prevalence of emergency caesarean delivery (*p* = 0.038), preeclampsia (*p* = 0.056) and lower educational level (*p* = 0.046) compared to breastfeeding women. 12 months after delivery, we observed a higher frequency of multiparity among breastfeeding women (*p* = 0.017) compared to non-breastfeeding. Overall, we found low disease activity in both groups at all registrations in the follow-up, and disease activity did not differ between the groups. More than 70% of both breastfeeding and non-breastfeeding women used hydroxychloroquine (HCQ).

**Conclusions:**

Breastfeeding rate in women with SLE was high six weeks postpartum. Multiparous women breastfed longer than primiparas. Disease activity, use of HCQ, and self-reported health data were comparable between the groups. Our data indicate that health professionals should encourage women with SLE to breastfeed.

## Background

Systemic lupus erythematosus (SLE) is a chronic systemic, inflammatory connective tissue disease characterized by idiopathic B-cell activation, formation and deposition of immune complexes and complement activation [1]. SLE can manifest in numerous ways and affect almost any organ of the body [2–5]. The disease often affects women of fertile age, and typically shows a relapsing – remitting disease course [6]. Pregnancy is a well-known and important trigger of disease exacerbations [7]. Both flares and severe disease manifestations like lupus nephritis, hypertension and/or presence of antiphospholipid antibodies, are associated with increased risk of adverse pregnancy outcomes [7]. Therefore, conception is recommended during periods of disease inactivity, and close monitoring by both rheumatologists and obstetricians is recommended [7]. The last two-thirds of pregnancy and the first year postpartum are associated with a higher risk of increased disease activity in SLE patients [7–9]. In recent years, pregnancy outcomes and disease activity during pregnancy have been studied, contributing to improved monitoring and treatment during pregnancy, leading to reduced risk of adverse pregnancy outcomes in SLE patients compared to previous reported risks [7, 10, 11].

An Italian study published in 2021 showed that initiation of breastfeeding in women with SLE was similar to initiation in the general population, however a higher proportion of the patients discontinued within three months [12]. The impact of the disease on initiation of breastfeeding is still not known. According to the Norwegian Central Bureau of Statistics (SSB, 2013), 97% of women in the Norwegian general population initiate breastfeeding and 30% are breastfeeding one year after delivery [13].

The aim of the study was to examine the frequency of breastfeeding, both full and partial breastfeeding, in women diagnosed with SLE at six weeks, six and 12 months postpartum. We compared breastfeeding and non-breastfeeding women in terms of age, parity, medical treatment, self-reported health data and disease activity.

## Methods

### Study design and data source

The current study was conducted with data from the RevNatus register [14], a Norwegian nationwide medical quality register designed for prospective follow-up of women with inflammatory rheumatic diseases from the time of planning a pregnancy until one year postpartum. The register provides data on demographic variables disease activity, medication, laboratory status, pregnancy outcome, self-reported health status and lactation. The register does not distinguish between full and partial breastfeeding. Data are recorded preconception, every trimester of the pregnancy in addition to six weeks, six and 12 months after delivery. In total, this comprises seven registrations. The register is operated by The Norwegian National Advisory Unit on Pregnancy and Rheumatic Diseases (NKSR). All patients above 16 years of age with a rheumatic diagnosis planning pregnancy are eligible for inclusion in RevNatus. The current study included women with a diagnosis of SLE verified by a rheumatologist according to the EULAR/ACR classification criteria. All together 133 pregnancies were registered. Pregnancies resulting in miscarriages, therapeutic abortions or stillbirths were excluded, thus resulting in a study population of 101 women and 114 pregnancies. In the current study we used data from inclusion in the register (pre-pregnancy or first trimester), six weeks, six and 12 months postpartum.

### Description of outcome variables

Prior to, or during each visit, patients were asked to report self-perceived degree of symptoms such as pain, fatigue and total disease burden. Remaining data were collected by healthcare workers through clinical examination and blood samples in conjunction with outpatient clinic controls. Demographic data included age, work status, educational level, parity, disease duration, body mass index (BMI), cigarette and snuff tobacco use, and exercise/activity level. Disease specific information on Visual Analogue Scale regarding pain and fatigue, Lupus activity Index in Pregnancy (LAI-P) [15] or SLE Disease activity Index (SLEDAI) [16] scores, C- reactive protein (CRP) level, serology with antinuclear antibodies (ANA) and anti-phospholipid (aPL) antibodies were also collected. Additionally, information on prior pregnancies (birth year, total pregnancy length and delivery method) was included. Demographic data, lactation status (breastfeeding yes/no) as well as medication use were registered at each visit. In our analysis, medications were grouped as hydroxychloroquine (HCQ), synthetic Disease Modifying Anti-Rheumatic Drugs (sDMARDs) and biologic DMARDs (bDMARDs). At the six-week visit, data relevant to pregnancy outcome were collected, including information on date of birth, date of term, prematurity (delivery prior to week 37), birth weight, sex of the infant, delivery method (vaginal or caesarean delivery), and occurrence of preeclampsia, eclampsia or HELLP (Hemolysis, Elevated Liver enzymes, Low Platelets) – syndrome during the pregnancy.

To assess disease activity, the verified composite measures LAI-P or SLEDAI were registered at each visit. In 2020, SLEDAI replaced LAI-P as the preferred method of assessing disease activity in the RevNatus database. Thus, both LAI -P and SLEDAI scores are included in our data. LAI-P is a modified version of the Lupus Acitivty Index (LAI) for use in pregnancy and scores disease activity on a scale from 0 to 26, using 15 parameters and dividing the data into four groups according to symptom severity. Scores vary from the minimum of 0 to a maximum of 2.6. Scores ≥ 2.2 or an increase in score by 0.25 is defined as high disease activity (flare) [9]. A *severe* exacerbation is defined as a flare involving the central nervous system, kidneys, lungs, conditions as vasculitis, myositis, hemolytic anemia, thrombocytopenia, and use of high doses of prednisolone/other immunosuppressive medications [17]. SLEDAI scores are calculated by evaluating 24 SLE specific disease domains, with a minimum score of 0 (“no disease activity”) and a maximum of 105 (“maximal disease activity). A score > 6 is considered as active disease requiring medical treatment [18].

Self-reported scores assessing pain, fatigue and total disease burden were performed at each visit using (VAS). The scale ranges from 0 to 100 and each domain is scored separately (0 = no pain/fatigue, 100 = extreme pain/fatigue). Thus, one could monitor changes in self-perceived experiences regarding symptom pressure using this as a measure on everyday functioning through the follow-up period [19].

### Statistical analysis

Statistical analyses were conducted using IBM SPSS Statistics Data Editor for Windows Version 27.0. A two-sided *p*-value ≤ 0.05 was considered statistically significant with no adjustments made for multiple comparison. Values are expressed as mean ± SD or median with interquartile range (IQR). Group comparisons were performed using Fisher´s exact test for categorical variables and independent samples t-test for continuous, normally distributed variables. For ordinal and continuous, non-normally distributed data, we used Mann Whitney U tests.

## Results

**Fig. 1:**
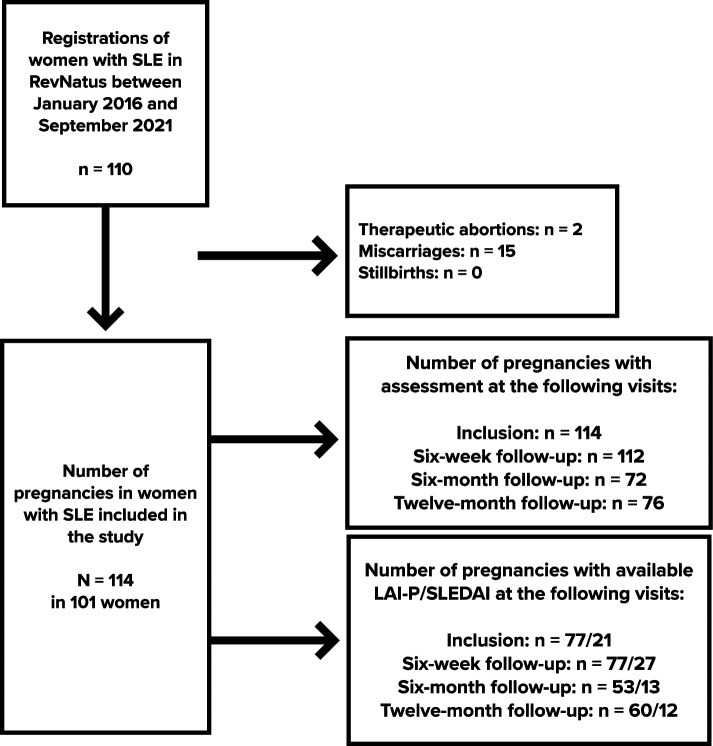
Flow chart of inclusion and exclusion of patients in the study

Figure 1 shows participants and exclusion data. Between January 2016 and September 2021, 129 pregnancies in 110 women with SLE were registered in the RevNatus database. However, 15 pregnancies not ending in live births were excluded. As demonstrated in Fig. 1, our study included 114 pregnancies in 101 women. Twin pregnancy occurred in one woman, and 13 women contributed with two pregnancies during the study period. As shown in Fig. 1, not all visits were attended, resulting in missing data. Mean number of postpartum visits per pregnancy was 2.3. On average, women contributing with two pregnancies attended fewer visits during their second pregnancy. Overall, the six-month follow-up had the lowest attendance with 69 women (61%).

Initiation rate of breastfeeding in our cohort was 91%. Six weeks after delivery 89 (78%) of women with SLE were breastfeeding. Six and 12 months postpartum, respectively 54% and 30% were breastfeeding. One year postpartum 11 primiparas (20%) were breastfeeding compared to 16 multiparas (46%). Multiparous women continued breastfeeding substantially longer than first-time mothers with SLE.

Six weeks postpartum, 13 primiparas did not breastfeed. Two of them had developed preeclampsia and three had premature deliveries (before week 37). Correspondingly, ten multiparas had stopped breastfeeding 6 weeks after delivery, where three women had developed both preeclampsia and had premature deliveries. Within 6 weeks postpartum, one woman had stopped breastfeeding due to initiation of medication incompatible with lactation. Both six and 12 months postpartum, five women had stopped breastfeeding due to medication changes.

Table 1 demonstrates patient background and disease-related characteristics collected at register inclusion. Patients were included when planning pregnancy or during the first trimester. Disease activity at inclusion was low in most patients, as well as CRP level. The maximum CRP value at inclusion was 38 mg/L.

**Table 1:** Patient background and disease-related characteristics collected at time of inclusion

Number of included women with SLE in the study	*N* = 114
Age mean (SD)	31.1 (4.7)
Age ≥ 35 yrs (%)	30 (26.3)
Parity n (%)	
0	64 (56.1)
1	34 (29.8)
≥ 2	16 (14.0)
Smoking / snuff tobacco	3 (2.6)
BMI kg/m^2^ n(%)	
Underweight (< 18.5 kg/m^2^)	4 (4.0)
Normal weight (18.5–24.9 kg/m^2^)	61 (61.6)
Overweight (≥ 25 kg/m^2^)	34 (34.5)
Regular physical activity (%)^a^	51.0
Educational levels (%)^b^	
Low	6.4
Intermediate	22.0
High	71.6
Working fulltime or part-time (%)	66.4
**Disease related characteristics**	
Disease duration in years mean (SD)	7.6 (5.5)
CRP mean (SD)	3.6 (5.0)
CRP value > 5 mg/L (%)	16.2
Anti-phospholipid antibodies present n (%)	30 (29.1)
ANA/ENA CTD screening positive n (%)	87 (81.3)
Inflammatory active disease n (%)^c^	
No /low disease activity	87 (88.8)
Moderate disease activity	10 (10.2)
High disease activity	0
**Medications**	
HCQ	85 (74.6)
sDMARDs^d^	34 (29.8)
bDMARDs^e^	1 (0.9)

*BMI* body mass index, *CRP* C-reactive protein, *ANA* anti-nuclear antibodies, *ENA* extractable nuclear antigens, *SD* standard deviation, *CTD* connective tissue disease, *HCQ* hydroxychloroquine, *sDMARDs* synthetic disease modifying anti-rheumatic drugs, *bDMARDs* biologic disease modifying anti-rheumatic drugs

^a^at least once weekly

^b^educational level: low = elementary school, medium = high school/vocational education, high = college/university

^c^measured by LAI-P/SLEDAI. Active disease: ≥ 2,2 (LAI-P) or > 6 (SLEDAI)

^d^sDMARDs included azathioprine, methotrexate, sulphasalazine and mycophenolate mofetil

^e^bDMARDs included belimumab, TNF-inhibitors and interleukin inhibitors

Table 2 shows breastfeeding rates six weeks, six months and 12 months postpartum and differences in breastfeeding rates between primi– and multiparas. Breastfeeding rates were comparable in primiparas and multiparas at the six-week visit. Significantly fewer primiparas were breastfeeding compared to multiparas 12 months postpartum.

**Table 2:** Proportion of breastfeeding primiparous and multiparous women with SLE 6 weeks, 6 and 12 months after delivery

Time of registrations n (%)	Primiparous *n* = 64	Multiparous *n* = 50	*p*-value*
6 weeks	50 (79.4)	37 (77.1)	0.62
6 months	24 (51.1)	20 (57.1)	0.82
12 months	11 (20.0)	16 (45.7)	0.05

^*^Comparison between groups with Fisher’s exact test

As shown in Table 3, mean age was comparable between breastfeeding and non-breastfeeding mothers six weeks after delivery. However, the proportion of women above the age of 34 years was larger in the non-breastfeeding group compared to the breastfeeding group. We found this difference to be statistically significant. Caesarean deliveries were more frequent among non-breastfeeding women compared to breastfeeding women.

**Table 3:** Differences in breastfeeding and non-breastfeeding in mothers with SLE six weeks after delivery

	**N**	**Breastfeeding** ***N* = 87**	**N**	**Non-breastfeeding** ***N* = 24**	***p*-value **^**a**^
Age, mean (SD)	86	31.5 (4.2)	24	32.9 (5.4)	0.18
Age ≥ 35 yrs (%)		20.9		45.8	0.02
BMI kg/m^2^ mean (SD)	75	24.2 (4.8)	21	25.5(6.0)	0.43
Overweight (> 25 kg/m) (%)		42.9		36.4	0.63
Disease duration yrs (SD)		7.5 (5.4)		7.2 (6.0)	0.67
Duration of pregnancy (weeks)	85	38.0	24	37.1	0.26
Birth before gestational week 37 n (%)	85	15 (17.6)	24	7 (29.2)	0.25
Birth weight (grams) mean (SD)	86	3133 (650)	24	2869 (974)	0.42
C- section total %	86	14	24	29.2	0.12
Emergency c-section (%)		5.8		20.8	0.04
Preeclampsia (%)	84	7.1	24	21.7	0.06
VAS scores (0–100 mm) median (IQR)					
Pain	65	10 (2–22)	17	10 (5–21)	0.67
Fatigue	65	31 (8–56)	17	41 (17–50)	0.74
Global score	65	15 (3–35)	17	22 (7–45)	0.35
CRP mg/L mean (SD)	77	2.3 (5.6)	24	3.4 (4.3)	0.13
Inflammatory active disease^b^ (%)	71	10.0	20	15.0	0.69
Regular physical activity^c^ (%)	47	48.9	19	42.1	0.79
Educational levels (%)	83		23		0.05
Low		3.6		13.0	
Middle		20.5		26.1	
High		75.9		60.9	
Working status (full or part-time) (%)	86		24		
Medications, n (%)	87		24		
HCQ		64 (73.6)		19 (79.2)	0.79
sDMARDs^d^		28 (32.2)		7 (29.2)	1.00
bDMARDs^e^		0		1 (4.2)	-

*BMI* body mass index, *CRP* C-reactive protein, *VAS* visual analog scale, *IQR* interquartile range, *c-section* caesarean section, *HCQ* hydroxychloroquine, *sDMARDs* synthetic disease modifying anti-rheumatic drugs, *bDMARDs* biologic disease modifying anti-rheumatic drugs, Educational levels: low = elementary school, medium = high school/vocational education, high = college/university

^a^group comparisons were performed using Fisher´s exact test for categorical variables and independent samples t-test for continuous, normally distributed variables. For ordinal and continuous, non-normally distributed data Mann Whitney U test was performed

^b^measured by LAI-P/SLEDAI. Active disease: ≥ 2,2 (LAI-P) or > 6 (SLEDAI)

^c^at least once weekly

^d^sDMARDs included azathioprine, methotrexate, sulphasalazine and mycophenolate mofetil

^e^bDMARDs included belimumab, TNF-inhibitors and interleukin inhibitors

CRP levels and disease activity were generally low in both groups six weeks postpartum. The highest CRP value measured at the six-week visit was 45 mg/L in one breastfeeding woman.

We did not observe any significant differences in disease activity or self-reported variables between breastfeeding and non-breastfeeding women six and at 12 months postpartum (Tables 4 and 5).

**Table 4:** Differences in breastfeeding and non-breastfeeding mothers with SLE 6 months after delivery

	**N**	**Breastfeeding** ***N* = 44**	**N**	**Non-breastfeeding** ***N* = 38**	***p*-value**^**a**^
Age ≥ 35 years, (%)	44	18.2	38	36.8	0.08
BMI (kg/m2), mean (SD)	36	25.6 (5.6)	26	24.3 (5.5)	0.26
Overweight (≥ 25 kg/m2)		17 (47.2)		9 (34.6)	0.44
Work status (full -or parttime), (%)	39	7.7	27	11.1	0.68
VAS scores (0–100), median (IQR)	27		21		
Pain		24 (8–40)		20 (0–49)	0.88
Fatigue		26 (3–50)		30 (5- 63)	0.62
Global score		12 (3–37)		25 (8–55)	0.23
Inflammatory active disease^b^ (%)	36	5.6	23	4.3	1.00
CRP value (mg/L), mean (SD)	37	2.30 (2.2)	26	2.7 (3.1)	0.81
Regular physical activity^c^ (%)	18	66.7	21	61.9	1.00
Medications, n (%)	44		38		
HCQ		34 (77.3)		28 (73.7)	0.80
sDMARD^d^		16 (36.4)		13 (34.2)	1.00
bDMARD^e^		0		1 (2.6)	-

*SD* standard deviation, *BMI* body mass index, *CRP* C-reactive protein, *VAS* visual analog scale, *IQR* interquartile range, *HCQ* hydroxychloroquine, *sDMARDs* synthetic disease modifying anti-rheumatic drugs, *bDMARDs* biologic disease modifying anti-rheumatic drugs

^a^group comparisons were performed using Fisher´s exact test for categorical variables and independent samples t-test for continuous, normally distributed variables. For ordinal and continuous, non-normally distributed data Mann Whitney U test was performed

^b^measured by LAI-P/SLEDAI. Active disease: ≥ 2,2 (LAI-P) or > 6 (SLEDAI)

^c^at least once weekly

^d^ sDMARDs included azathioprine, methotrexate, sulphasalazine and mycophenolate mofetil

^e^bDMARDs included belimumab, TNF-inhibitors and interleukin inhibitors

**Table 5:** Differences in breastfeeding and non-breastfeeding mothers with SLE 12 months after delivery

	**N**	**Breastfeeding** ***N* = 27**	**N**	**Non-breastfeeding** ***N* = 63**	***p*-value**^**a**^
Age ≥ 35 years, (%)	27	22.2	63	28.6	0.61
BMI (kg/m2), mean (SD)	25	24.5 (4.0)	40	23.9 (5.1)	0.34
Overweight (≥ 25 kg/m2), (%)	25	44.0	40	33.3	0.43
Working status (full- or parttime), (%)	27	40.7	45	48.9	0.63
VAS scores (0–100), median (IQR)	23		33		
Pain		15 (1–61)		18 (4–26)	0.57
Fatigue		33 (7–73)		30(10–51)	0.75
Global score		13 (1–24)		25 (10–41)	0.06
Inflammatory active disease* (%)	21	9.5	28	21.4	0.44
CRP value (mg/L), mean (SD)	26	2.50 (4.5)	42	2.48 (3.1)	0.18
Regular physical activity** (%)	18	38.9	32	53.1	0.39
Medications, n (%)	27		63		
HCQ		22 (81.5)		45 (71.4)	0.43
sDMARD***		10 (37.0)		20 (31.7)	0.63
bDMARD****		0		1 (1.6)	-

*SD* standard deviation, *BMI* bodymass index, *CRP* c-reactive protein, *VAS* visual analog scale, *IQR* interquartile range, *HCQ* hydroxychloroquine, *s-DMARD* synthetic disease modifying anti-rheumatic drugs, *bDMARDs* biologic disease modifying anti-rheumatic drugs

^*^measured by LAI-P/SLEDAI. Active disease: ≥ 2,2 (LAI-P) or > 6 (SLEDAI)

^**^at least once weekly

^***^sDMARDs included azathioprine, methotrexate, sulphasalazine and mycophenolate mofetil

^****^bDMARDs included belimumab, TNF-inhibitors and interleukin inhibitors

^a^group comparisons were performed using Fisher´s exact test for categorical variables and independent samples t-test for continuous, normally distributed variables. For ordinal and continuous, non-normally distributed data Mann Whitney U test was performed

## Discussion

Our study based on data from the Norwegian quality register RevNatus, showed that breastfeeding rates in 101 women with SLE six weeks, six and 12 months after delivery, were 78%, 54% and 30%, respectively. Factors positively associated with breastfeeding were younger maternal age and multiparity. Only delivery by emergency caesarean section was associated with non-lactation.

The present study is currently the largest prospective study on breastfeeding in SLE. According to a nationwide survey performed by the Norwegian Central Bureau of Statistics (SSB) in 2013, 97% of Norwegian mothers initiated breastfeeding in the general population [13]. This is slightly higher than in our cohort (91%).

Our study showed an association between duration of lactation and parity. This has been shown in other studies as well, and may be explained by an association between prior positive breastfeeding experience and breastfeeding duration [20, 21]. Primiparity has been identified as a risk factor for delayed lactogenesis [22]. Breastfeeding initiation and duration have been found to increase with the age of the mother [23]. However, in our cohort, we found that women older than 34 years discontinued breastfeeding earlier than younger women.

Although knowledge on SLE and breastfeeding is sparse, a few studies on the topic have been published. The analysis of Noviani et al., assessing 51 pregnancies in women from the Duke Autoimmunity in Pregnancy Registry during the period of 2008 to 2013, showed that approximately 50% initiated breastfeeding [24]. Factors associated with breastfeeding were low disease activity after delivery, full term delivery and planning of breastfeeding early in the pregnancy. However, the American study included a larger proportion of participants with high disease activity compared to our study population.

Also, a study from Argentina showed that among 31 mothers with SLE and 31 non-SLE mothers, 19% of the patients did not initiate breastfeeding compared to 6% of the controls [6]. The average duration of breastfeeding in SLE mothers was six months, and 12 months in non-SLE mothers. The Argentinian study was retrospective and based on self-reported data. The patients had pregnancies subsequent to their diagnosis of SLE. Time from delivery to reporting data was in average 5 years for patients and controls.

Opposite, an Italian study including 43 SLE patients with 57 pregnancies, and with self-reported data on breastfeeding during 2008 to 2019, showed no significant difference in initiation of breastfeeding between SLE mothers and women in the general Italian population [12]. This is comparable to our results. However, the median time of breastfeeding was three months among the patients which is substantially shorter than in the Norwegian study.

The disease should have stayed inflammatory inactive for at least six months in women with SLE planning pregnancy [7]. Although pregnancy is a well-known trigger for increased SLE disease activity [7], a previous Norwegian study showed low or no disease activity at conception and during pregnancy in most SLE women [9]. This correlated well with our results, as all participants had low disease activity scores at inclusion of RevNatus. Skorpen et al. found that disease activity was significantly higher six and 12 months postpartum [9] compared to time of conception. In contrast, our study showed an overall low postpartum disease activity, with a mean LAI-P score of 0.15 and a mean SLEDAI score of 1.4 The extensive use of HCQ among our patients (75%) may be one explanation for the very few flares observed postpartum. HCQ has been recommended for use in pregnancy and during lactation by the ACR (American Collegue of Rheumatology) organization [25].

A higher proportion of elective and emergent caesarian deliveries in non-breastfeeding women with SLE has previously been reported [12]. In our study, only delivery by emergency caesarean section was positively associated with non-breastfeeding at six weeks after delivery. Positive associations between initiation and duration of breastfeeding and term delivery have also been demonstrated in a previous study [6]. We did not find a significant association between prematurity and non-breastfeeding or cessation of breastfeeding. However, analyzing the pregnancies ending in emergency caesarean delivery (*n* = 10), 50% were born preterm. A previous study has shown that up to 80% of preterm mothers have difficulties establishing sufficient milk supplies and initiating lactation compared to mothers delivering at term [26]. This corresponds to our data, showing that 80% of mothers of preterm infants delivered by emergency caesarean section had discontinued breastfeeding within six months after birth. Acute surgical delivery may lead to significant maternal and fetal stress, which are found to reduce oxytocin release. This in turn may compromise lactogenesis [22, 27]. Not being able to sufficiently supply your child with breastmilk, could possibly worsen the stress response thus reducing lactogenesis even further. These factors may lead to early cessation of breastfeeding.

A limitation of our study was that we were not able to distinguish between full and partial breastfeeding. However, traditionally, Norwegian women do full breastfeeding, also women with rheumatic diseases, during the first three to six months after delivery. In Norwegian maternity wards, all mothers are encouraged to initiate breastfeeding. However, the length of stay in maternity wards are substantially shorter than previous years. This results in shorter follow-up time in hospital, which may lead to early cessation of breastfeeding in some women. Another limitation was the switch of preferred disease activity scoring index, from LAI-P to SLEDAI. At inclusion 79% of the pregnancies were assessed using LAI-P. The scoring systems emphasize and account for different elements, thus, the proportion of exacerbations at different time points could be incorrectly estimated. A third limitation is related to the amount of missing data. This applies especially to the six-month visit which had the lowest attendance (61%), leading to large amounts of missing data at this point. Also, participants did not always complete the digital self-registration prior to each visit, resulting in missing information. Large quantities of missing data pose a risk of type 2 errors. Non-significant data should therefore be interpreted with caution.

The main advantage of our study is the prospective design, allowing us to monitor the participants over time in a public health care system available to all the population, securing more accurate and unbiased registrations of disease activity and quality of life. The diagnoses of SLE were confirmed by an experienced rheumatologist and according to the ACR/EULAR classification criteria. Additionally, by including missing data from subjects who refrained from visits at certain time points, we have exploited all available data in our analyses.

## Conclusion

In the current study, the proportion of breastfeeding women with SLE were comparable with the proportion of breastfeeding in the general Norwegian population. Breastfeeding was positively associated with multiparity, younger maternal age and non-operative delivery methods. There were few or no disease-related differences between breastfeeding and non-breastfeeding mothers in SLE six and 12 months postpartum. Extensive use of HCQ has likely contributed to lower disease activity postpartum, and may have resulted in a higher proportion of patients initiating breastfeeding than reported in prior studies.

## Data Availability

The datasets used and analyzed during the current study are available from the corresponding author on reasonable request.
